# Pyrimidine 2,4-Diones in the Design of New HIV RT Inhibitors

**DOI:** 10.3390/molecules24091718

**Published:** 2019-05-02

**Authors:** Roberto Romeo, Daniela Iannazzo, Lucia Veltri, Bartolo Gabriele, Beatrice Macchi, Caterina Frezza, Francesca Marino-Merlo, Salvatore V. Giofrè

**Affiliations:** 1 Dipartimento di Scienze Chimiche, Biologiche, Farmaceutiche ed Ambientali, Università di Messina, Via S.S. Annunziata, 98168 Messina, Italy; 2 Dipartimento di Ingegneria, Università di Messina, Contrada Di Dio, 98166 Messina, Italy; diannazzo@unime.it; 3 Dipartimento di Chimica e Tecnologie Chimiche, Università della Calabria, Via P. Bucci 12/C, 87036 Arcavacata di Rende, Italy; lucia.veltri@unical.it (L.V.); bartolo.gabriele@unical.it (B.G.); 4 Dipartimento di Scienze e Tecnologie Chimiche, Università di Roma “Tor Vergata”, 00133 Roma, Italy; macchi@med.uniroma2.it (B.M.); catafrezza@hotmail.it (C.F.); 5 IRCCS Centro Neurolesi “Bonino-Pulejo”, 98124 Messina, Italy; fmarino@unime.it

**Keywords:** reverse nucleosides, Pyrimidine-2,4-dione derivatives, HIV RT inhibitors, biological activity, molecular docking

## Abstract

The pyrimidine nucleus is a versatile core in the development of antiretroviral agents. On this basis, a series of pyrimidine-2,4-diones linked to an isoxazolidine nucleus have been synthesized and tested as nucleoside analogs, endowed with potential anti-HIV (human immunodeficiency virus) activity. Compounds **6a**–**c**, characterized by the presence of an ethereal group at C-3, show HIV reverse transcriptase (RT) inhibitor activity in the nanomolar range as well as HIV-infection inhibitor activity in the low micromolar with no toxicity. In the same context, compound **7b** shows only a negligible inhibition of RT HIV.

## 1. Introduction

Continuous efforts in the search of new antiviral agents are a consequence of the urgent demand for new therapeutic agents in which an improved biological activity against viruses is matched with low toxicity towards host cells. In this context, over recent years the ever-growing development of new anti-retroviral drugs has turned the prognosis of human immunodeficiency virus (HIV)-1 infection from a terminal into a chronic disease. In particular, highly active antiretroviral therapy (HAART) has significantly changed the progression and outcome of infection with HIV-1 [1,2,3,4,5]. HAART comprises four major classes of drugs: nucleoside reverse transcriptase (RT) inhibitors (NRTIs), non-nucleoside RT inhibitors (NNRTIs), protease inhibitors (PIs), and integrase INST inhibitors (INSTIs).

In order to overcome some of the limits and side effects of the current treatment regimes, new therapeutic approaches have been investigated for HIV treatment, such as the use of antagonists of CCR5, a receptor involved in the virus entry. In this context, the m-Tor inhibitor rapamycin provides a potential strategy to inhibit HIV, especially in patients with drug resistant HIV strains [6,7,8,9,10].

In spite of the consistent benefits for HIV-1 infected patients undergoing antiretroviral therapy, a complete immune reconstitution is usually not achieved. Actually, antiretroviral therapy may be frequently accompanied by immunological unresponsiveness, persistent inflammatory conditions and inefficient cytotoxic T-cell response [11,12,13,14]. Accordingly, structurally novel HIV inhibitors, particularly those with a distinct mechanism of action, could add significantly to the existing HAART repertoire, overcoming several side effects linked to the continuous exposition to antiretroviral drugs and the higher risk of developing co-morbidities, such as cardiovascular, metabolic and neurological disease [15,16,17,18].

The reverse transcription (RT) of the viral single-stranded (+) RNA genome into double-stranded DNA plays a basic role in the replication of HIV. Due to this essential step in the viral life cycle, RT constitutes the target of numerous anti-HIV drugs that are key components of HAART [19,20,21,22,23,24,25].

Two classes of drugs, NRTIs and NNRTIs, have been reported as inhibitors of RT [26,27,28,29,30,31]. NRTIs act as DNA chain terminators, while NNRTIs bind to a hydrophobic pocket close to the RT active site and inhibit the enzyme activity by mediating allosteric changes in the RT conformation, thus causing a distortion in the arrangement of the catalytic active site aspartyl residues [32,33,34,35,36,37]. However, in spite of the efficiency of some NRTIs and NNRTIs, the rapid emergence of multidrug-resistant mutants has promoted the research of new anti-HIV agents with significantly improved drug resistance profiles [38,39,40,41,42,43,44].

In particular, NNRTIs have received a lot of attention because of their favorable potency and low cytotoxicity. So far, five NNRTIs have been approved for AIDS treatment, and about 50 classes of structurally diverse NNRTIs are being widely investigated [45,46,47,48]. 

**Figure 1:**
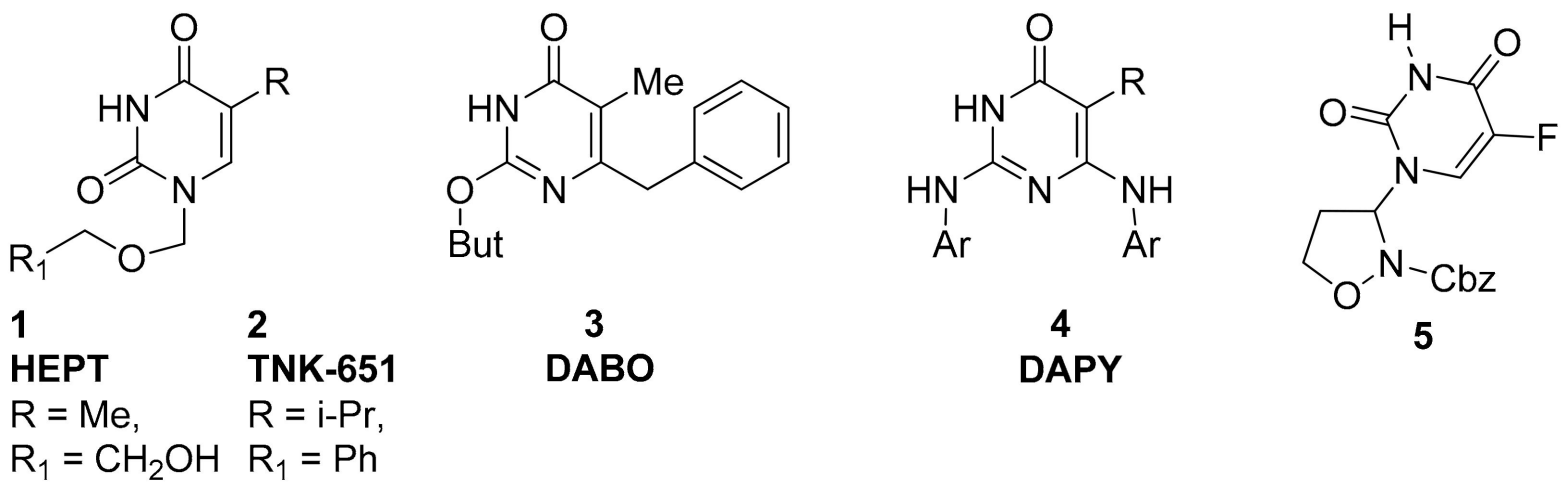
Modified pyrimidines as non-nucleoside reverse transcriptase inhibitors (NNRTIs).

In this research field, the pyrimidine nucleus represents a versatile chemical core in the design of many antiretroviral agents acting as NNRTIs: Modified pyrimidines constitute the backbone of many non-nucleoside reverse transcriptase inhibitors. Diaryl-pyrimidines (DAPYs, **1** [49,50,51]) and their derivatives, 2-alkoxy-6-benzyl-3,4-dihydro-4-oxopyrimidine (DABO, **2** [52,53]), 1-[(hydroxyethoxy)-methyl]-6-(phenyl-sulfanyl)thymine (HEPT; **3** [54,55]) and its analogue (TNK-651; **4**) (Figure 1) are efficient NNRTIs that—through binding at the allosteric, non-nucleoside binding pocket (NNIBP) of RT—prevent the conformational transition needed for the formation of a productive polymerase–RNA complex [56].

Recently, we have reported the synthesis and biological activity of the new class of 3′-pyrimidinyl isoxazolidines **5**, the truncated reverse isoxazolidinyl nucleosides (TRINs), as HEPT analogues [57]. When tested in vitro for their biological activity, compound **5** showed a nearly complete inhibition of avian myeloblastosis virus (AMV) RT and HIV RT in the nanomolar range, with weak cytotoxicity towards human cells.

Docking data indicate that an important role could be played by the substituent at the C-5 position. On this basis, according to the exploration of chemotypes amenable to the construction of new compounds endowed with significantly improved drug resistance profiles, compared with the first generation NNRTIs, we have been interested in the evaluation of a series of compounds, amenable to pyrimidine-2,4-dione scaffold, where structural elaboration, both at the level of the nucleobase and the isoxazolidine unit, have been performed in order to improve the stabilizing interactions suggested by docking data. 

**Figure 2:**
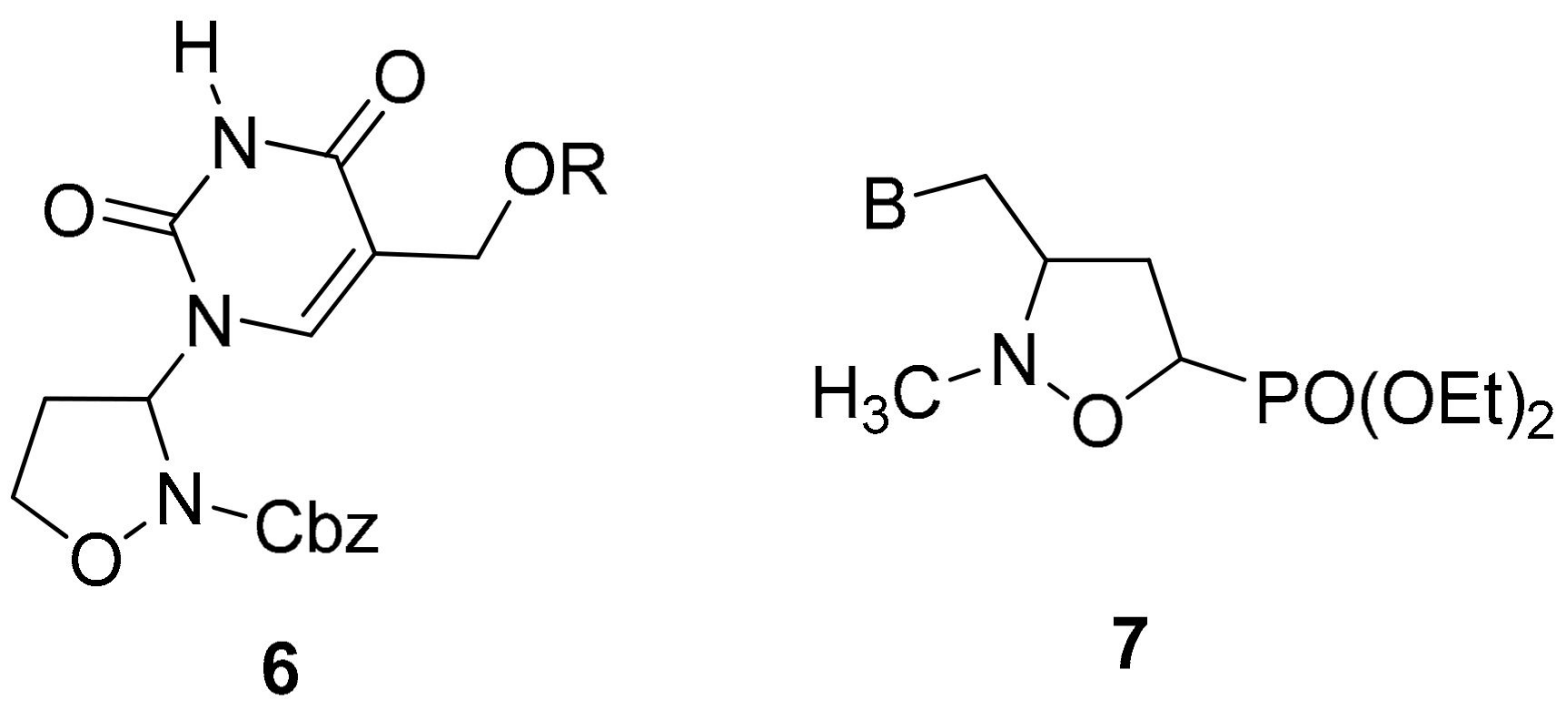
New synthesized modified pyrimidines as potential antiviral agents.

We report here the synthesis and biological evaluation of compound **6**, where the substituent at C-5 has been replaced with an ethereal unit in order to favor hydrogen bond interactions and to improve hydrophobic interactions with the hydrophobic residues in the RT pocket [57]. In the same context, according to our program targeted to the discovery of new compounds able to interfere with viral replication of HIV, the redesign of N-1 moiety led to phosphonated reverse homo-*N*,*O*-nucleosides **7**, characterized by the presence of a phosphonic group at C-4′, where a methylene linker is incorporated between the nucleobase and the isoxazolidine ring: The absence of the natural N-glycosidic bond should result in a greater metabolic stability of these compounds due to their inertness towards the hydrolytic activity of cellular phosphorylases (Figure 2). The insertion of the phosphonic group at C-4′ is supported by the consideration that phosphonated *N*,*O*-nucleosides should be able to bypass the initial selective enzymatic monophosphorylation step and overcome the instability of nucleotides toward phosphodiesterases [58].

Antiviral assays showed that some of the new synthesized pyrimidin-2,4-dione derivatives, in particular some of those amenable to derivatives **6**, inhibited HIV RT activity in the nanomolar range and HIV-1 infection in the low micromolar range, without cytotoxicity, at concentrations up to 100 μM.

## 2. Results and Discussion

### 2.1. Chemistry

**Scheme 1:**
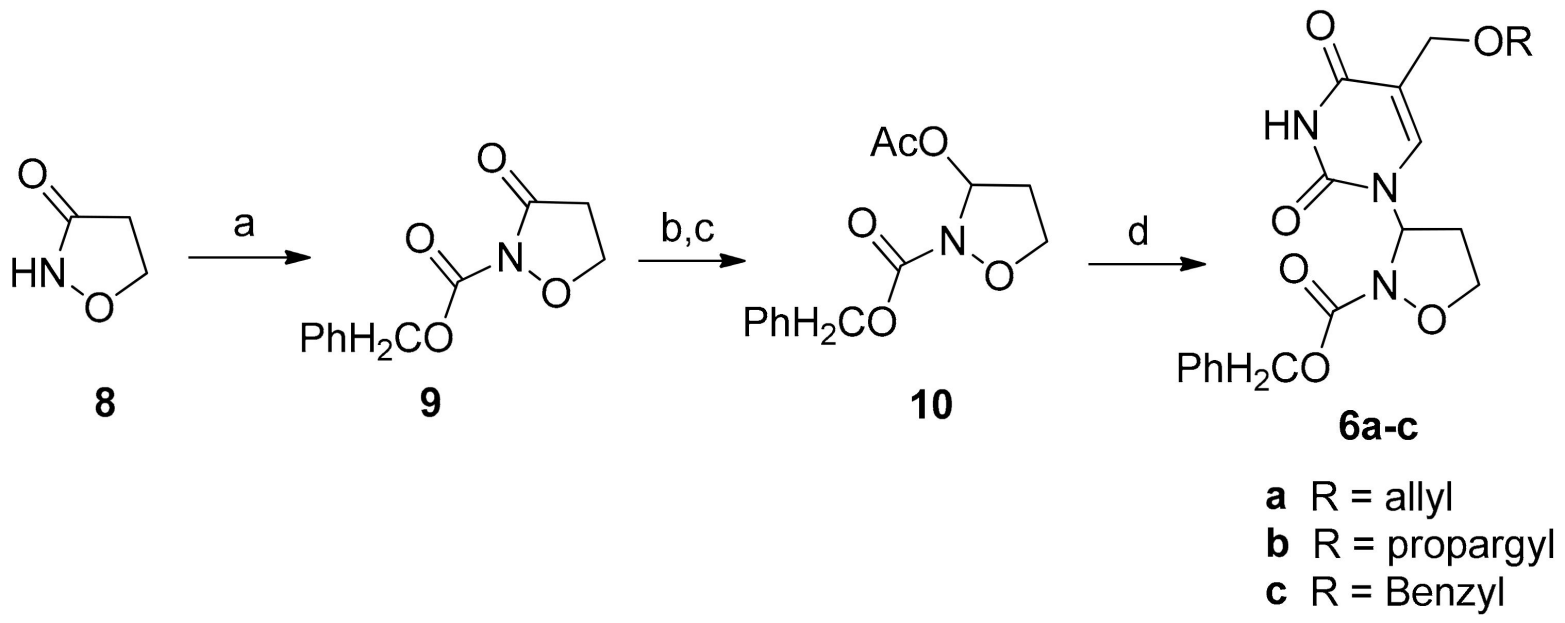
Synthesis of 3′-pyrimidinyl isoxazolidines **6a**–**c**: (a) ETA, PhOCH_2_C(O)Cl, DMF, room temperature; (b) Cp_2_Zr(H)Cl) (4 equiv), THF, SiO_2_, 1 h, room temperature; (c) Ac_2_O, ETA, DCM, room temperature, 6 h; (d) 5-substituted uracil **11a**–**c**, BSA, TMSOTf, CH_3_CN, r.t., 4 h.

The synthesis of isoxazolidinyl pyrimidines **6a**–**c** is described in Scheme 1. The approach, adapted from the route reported for the synthesis of compound **5** [57], starts from the isoxazolidin-3-one **8** [59,60], which was reduced with Cp_2_Zr(H)Cl to the corresponding azahemiacetal **9**, and then treated with Ac_2_O with the formation of the acetyl derivative **10** (Scheme 1).

The subsequent coupling of **10** with 5-substituted uracils **11a**–**c**, prepared by reaction of 5-hydroxymethyluracil with allylic, propargylic and benzylic alcohols, respectively, in conc. HCl at reflux, afforded the isoxazolidinyl pyrimidines **6a**–**c** in good yields.

**Scheme 2:**
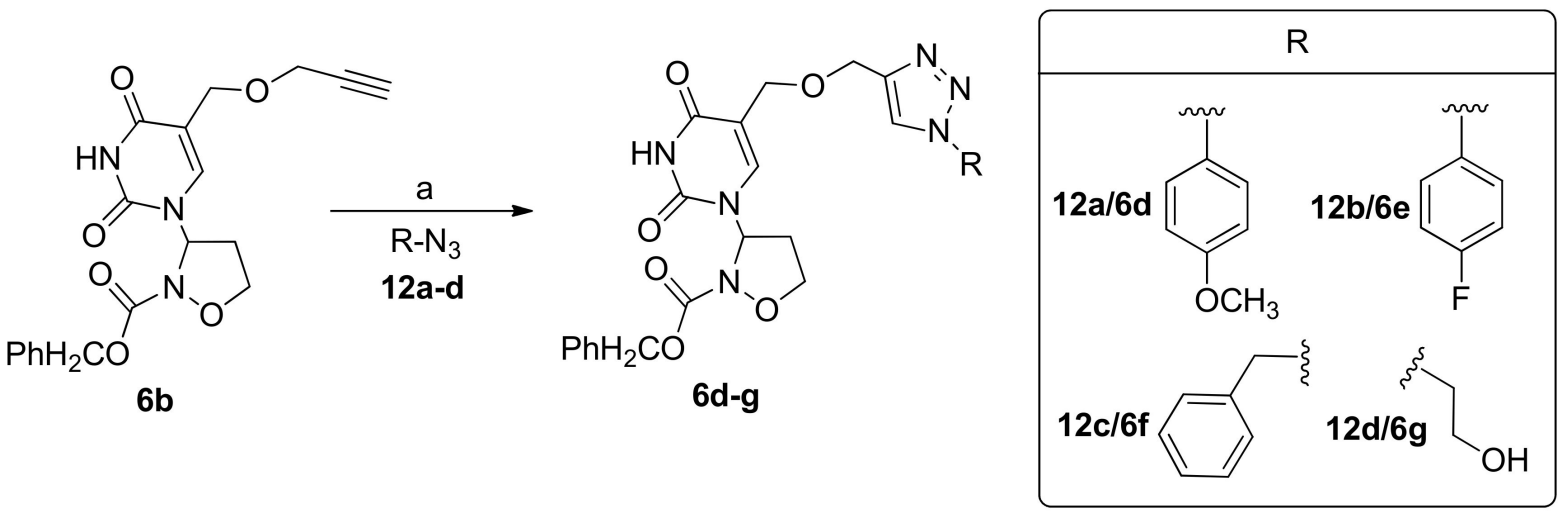
Synthesis of 3′-pyrimidinyl isoxazolidines **6d**–**g**: (a) CuSO_4_·5H_2_O, sodium ascorbate, Et_3_N, room temperature, 4 h.

According to the data suggested by docking experiments, which indicate that the presence of an aromatic unit in the ethereal chain at C-5 could improve the potential biological activity as NNRTI of the synthesized compounds, we also investigated the possible role of the replacement of the phenyl ring of compound **6b** with an heteroaromatic system. For this aim, compound **6c** was reacted with azides **12a**–**d** (Scheme 2). Derivatives **6d**–**g** were easily obtained in very good yields.

**Scheme 3:**
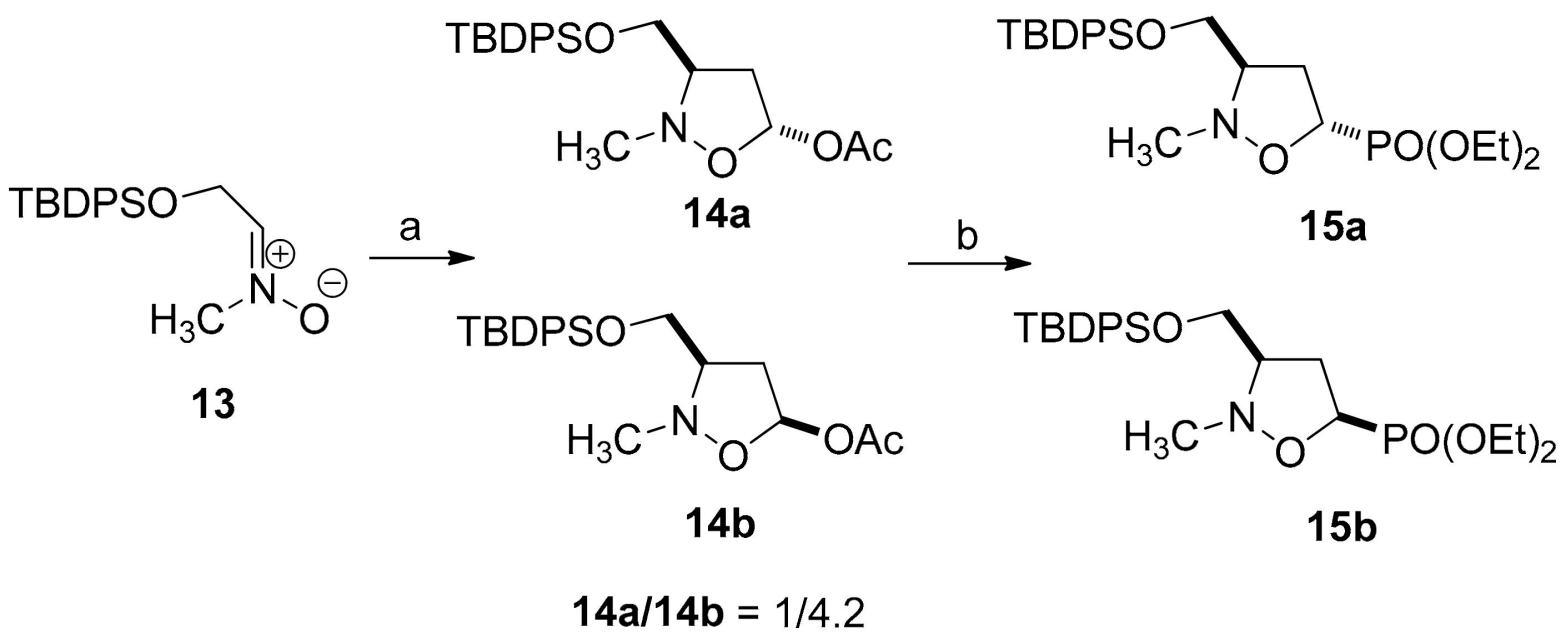
Synthesis of phosphonated reverse homo-*N*,*O*-nucleosides **7a**–**d**: (a) vinyl acetate, CH_2_Cl_2_, 4 h, room temperature; (b) P(OEt)_3_, TiCl_4_, CH_2_Cl_2_, 4 h, −30 °C; (c) TBAF, CBr_4_, THF, room temperature, 6 h; (d) nucleobase, Cs_2_CO_3_, DMF, 95 °C, 4 h, room temperature.

Phosphonated reverse homo-*N*,*O*-nucleosides **7** have been described in literature and tested as antitumoral agents [61]. Here, we report a different synthetic route which proceeds with better yields and allows the obtainment of pure α and β anomers. The new designed process combines a 1,3-dipolar cycloaddition, leading to the isoxazolidinyl system, and an Arbuzov reaction to insert the phosphonic group at C-5. Finally, a nucleosidation process introduces the pyrimidine nucleobase at C-3 position. Thus, C-[(tert-butyldiphenylsilyl)oxy]-*N*-methylnitrone **13** [62,63], as an isomeric E/Z mixture (1:5), was reacted with vinyl acetate to give with a good stereoselectivity the epimericisoxazolidines **14a** and **14b**, in a relative ratio 1:4.2 (global yield 90%). Compounds **14a** and **14b** were separated by flash chromatography; the phosphonate group was then introduced by a Lewis acid catalyzed Arbuzov reaction which afforded derivatives **15a** and **15b** (80% yield) (Scheme 3).

**Scheme 4:**
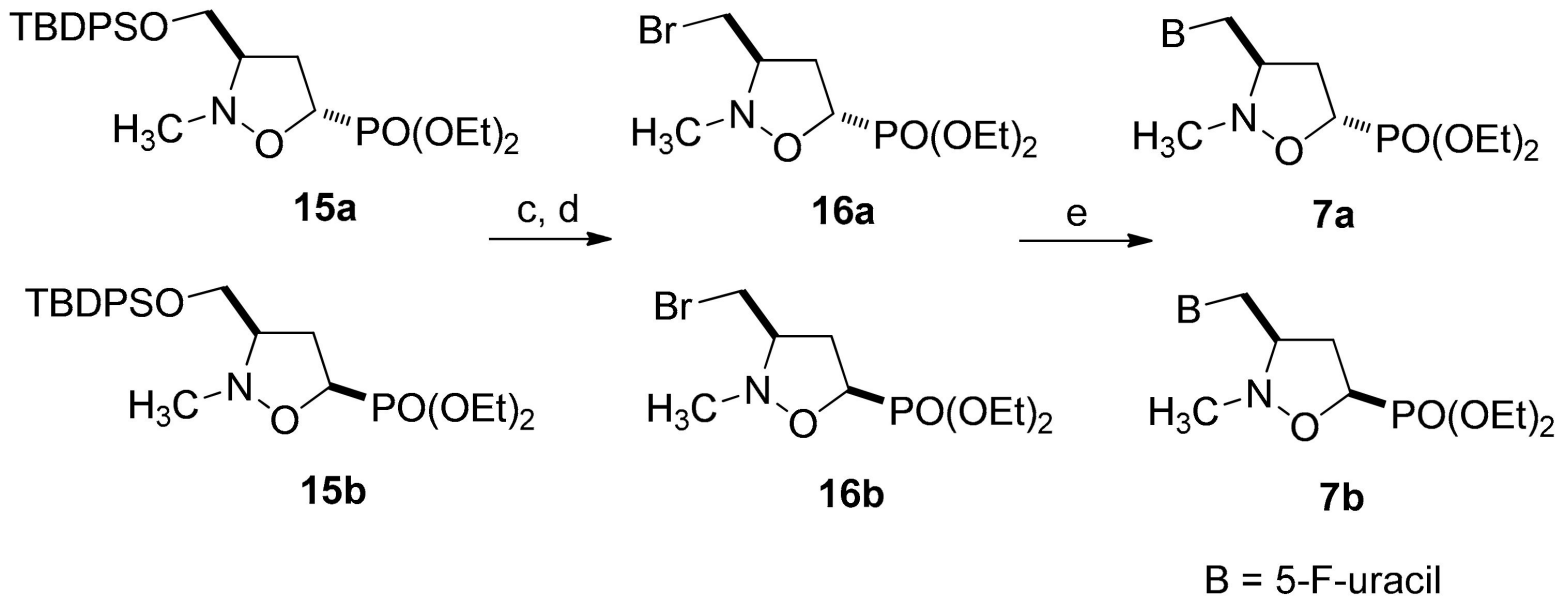
Synthesis of phosphonated reverse homo-*N*,*O*-nucleosides **7a**,**b**: (a) vinyl acetate, CH_2_Cl_2_, 4 h, room temperature; (b) P(OEt)_3_, TiCl_4_, CH_2_Cl_2_, 4 h, −30 °C; (c) TBAF, THF, 6 h, room temperature; (d) CBr_4_, THF, room temperature, 6 h; (e) nucleobase, Cs_2_CO_3_, DMF, 95 °C, 4 h, room temperature.

Compounds **15a** and **15b** were treated with TBAF in THF and subsequently converted into the corresponding bromides **16a** and **16b** (global yield 65%). The subsequent coupling with fluorouracil, in the presence of Cs_2_CO_3_ in DMF at 95 °C, completed the reaction route, affording the phosphonated reverse homo-*N*,*O*-nucleosides **7a**,**b** (Scheme 4).

### 2.2. Biological Activity

Compounds **6a**–**g** and **7a**,**b** were preliminarily tested in vitro for their cytotoxicity (CC_50_) towards MOLT-3 cells, a human T-lymphoblastoid cell line, through the MTS assay. Nevirapine (NVP), Rilpivirine (RPV) and Efavirenz (EFV) have been used as reference controls. Cytotoxicity against these cells was not observed with any of the **6a**–**c** and **7** analogues at concentrations up to 1000 µM. Conversely, a 50% cytotoxicity was observed for compounds **6d**–**g** (Table 1) at concentrations of 594 ± 0.8, 552 ± 3, 670 ± 1, 579 ± 2 µM, respectively. Nevirapine (NVP) showed cytotoxicity at concentrations higher than 1000 µM.

The antiretroviral activity of all the synthesized compounds was tested by a cell-free RT inhibitory assay, in dose-response fashion [64,65,66]. In particular, the RT inhibitory activity of the new compounds along with that of the reference antiviral compound, NVP, were tested versus HIV-1 or avian myeloblastosis virus (AMV) reverse transcriptase. The results reported in Table 1 show that reverse homo-*N*,*O*-nucleosides **7**, as well as pyrimidin-2,4-dione analogues containing an heteroaromatic system (**6d**–**g**) in the ethereal chain at the C-5 position, did not show any antiretroviral activity in the adopted conditions at concentrations up to >1 mM. Conversely, compounds carrying an allyl or a benzyl unit at C-5 (**6a** and **6c**) showed strong inhibition against HIV RT, but not AMV RT, with activities in the low nanomolar range. The highest inhibitor activity was evidenced by compound **6c** with an MRTIC value of 0.01 µM, not far from that of nevirapine (NVP). The good anti-HIV RT activity exerted by compound **6c** represents a significant starting point for future in-depth investigations regarding its in vitro and in vivo anti-HIV activity, toxicity, and oral bioavailability. Moreover, the reported data further confirm the relevance of pyrimidine nucleus as the chemical core in the design of new anti-HIV agents with an improved pharmacological profile. It is noteworthy that of the series of compounds **6a**–**c**, **6b** (characterized by the presence of a propargyl group) showed the lowest HIV-1 RT inhibitory effect.

We then focused our attention on compounds (**6a**–**c**) to check whether they directly inhibited HIV infection, through a recently developed cell infection assay [65]. For comparison, compound **6g** and NVP were also assayed. The assay consisted of infection with HIV-I infectious molecular clone NL4-3 (HIVNL4-3) of CEM human lymphoblastoid cells stably transfected with a plasmid of the green fluorescence protein under the control of HIV-LTR (CEM-GFP). CEM-GFP were unexposed or exposed to several concentrations of compounds or to NVP for 2 h at 37 °C in a humidified atmosphere supplemented with 5% CO_2_. Following infection with HIVNL4-3, cells were subjected to spinoculation and incubated for 3 h at 37 °C. After centrifugation, the excess of virus was removed and the compounds were re-added. Infection was determined by assessing GFP expression through flow cytometry analysis from 5000 events per sample, after 72 h of incubation. The results in Table 2, referring to one representative experiment of two performed with similar results, show that **6b** was endowed with a low IC50, not far from that of NVP. Compounds **6a** and **6c** proved inhibitory in a range from 10 to 15 µM. Interestingly, toxicity of compound **6b** towards uninfected CEM-GFP was even lower than that of NVP, ensuring a good selectivity index. Thus, the anti-HIV activity exerted by some of the compounds under investigation represents a significant starting point for future in-depth investigations regarding their actual antiretroviral potential. Moreover, the reported data further confirm the relevance of the pyrimidine nucleus as chemical core in the design of new anti-HIV agents with improved pharmacological profile. Noteworthy, in the series of compounds **6a**–**c**, **6b**, characterized by the presence of a propargyl group, showed the more potent inhibitory effect towards HIV-1 infection in a cell assay, while **6c**, characterized by a benzyl group, exhibited the lowest value of HIV-RT inhibition in a cell-free assay.

As previously reported [57], the RT inhibition exhibited by the tested compounds **6a**–**c** may be explained by an allosteric mechanism of action. In fact, these compounds were able to inhibit RT activity without any conversion by phosphorylation into the corresponding triphosphate forms. Thus, these compounds should not act as competitive inhibitors of RT with respect to the nucleotide substrate, but inhibition of the polymerase activity would occur before elongation, presumably by preventing the conformational transition needed to form a productive polymerase–RNA complex. 

**Figure 3:**
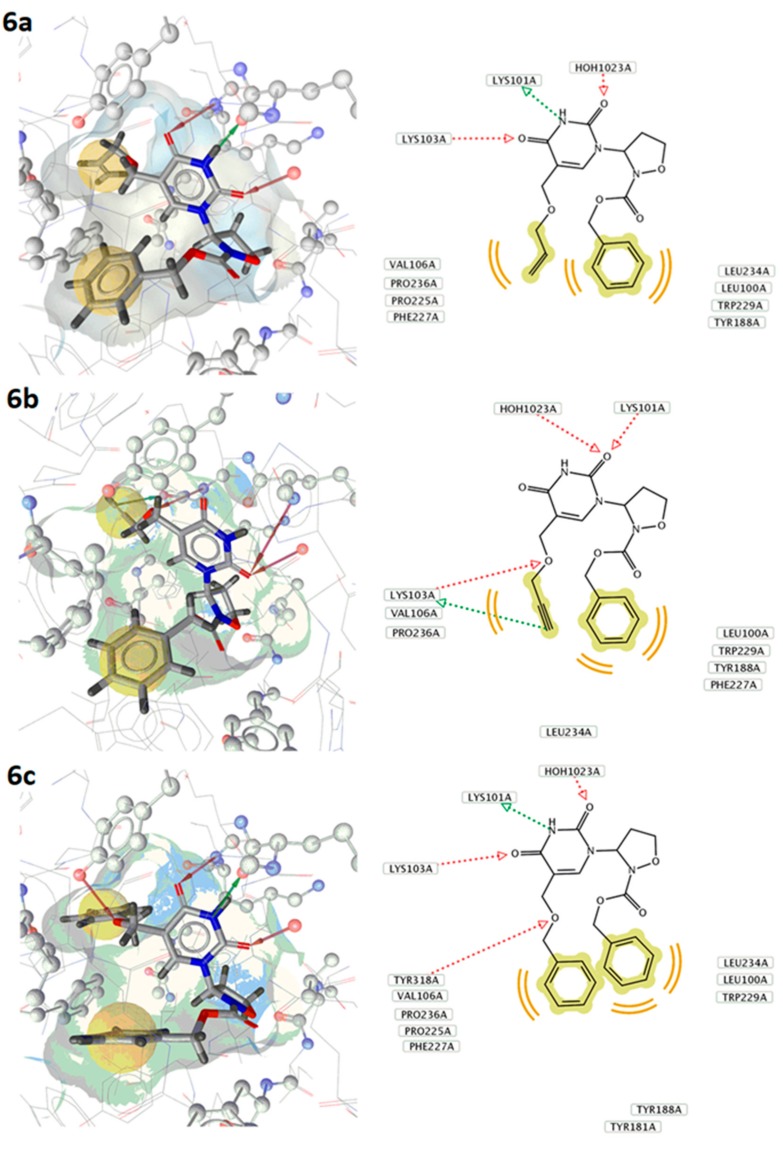
Docking interactions for compounds **6a**, **6b** and **6c**.

The biological activity observed for compounds **6a**–**c** has been rationalized on the basis of docking data [57] (see Supplementary Materials). Starting from the X-ray coordinates of the TNK-651–HIV-1 RT complex (PDB code: 1RT2), derivatives **6a**–**c** were docked into the wild-type HIV-1 RT non-nucleoside binding pocket (NNIBP); the binding free energies (ΔG_b_) are in the range of –7.45 to –8.51 kcal/mol (Table 3). The binding mode of compounds **6a**–**c** shows common interactions with the NNBP of RT (Figure 3). In particular, the aromatic ring of compound **6a** was found in close contact with Tyr188 and Trp229, thus allowing π-π type interactions. Additional hydrophobic contacts were established with the Leu234, Leu100 and Trp229, while the nucleobase interacted with the Lys101 by the NH group, which acts as a donor of hydrogen bond, and a polar interaction was exerted by the carbonyl group at C-2, which was oriented towards the water exposed in proximity to the positively charged ammonium group of Lys101. Furthermore, the presence of the allyl unit improves hydrophobic and π-π interactions with Pro225, Pro236, Val106 and Phe227. The aromatic ring of compound **6b** shows π-π interactions with Phe227, Tyr188 and Trp229; additional hydrophobic contacts are established with the Leu100, Val 106 and Pro236. 

Contrary to the effect exerted by the allyl unit in compound **6a**, the propargyl group of **6b** pushes the pyrimidine nucleus away from the Lys101 residue, leading to the loss of the hydrogen bond with pyrimidine NH. 

The docking of **6c**, the most active compound, confirms that the benzyl ethereal group at C-5 contributes to the stability of the resulting inhibitor–RT complex, leading to a favorable stabilizing interaction with Tyr318 residue at the allosteric site of RT-HIV, by the formation of a hydrogen bond. Moreover, the benzyl unit improves π-π interactions with Phe227 and additional hydrophobic contacts with Val106, Pro236 and Pro225; the nucleobase shows hydrogen bonds with Lys101 and Lys103 by NH and carbonyl group at C4, respectively.

In contrast with our expectations, the replacement of the phenyl ring in **6c** with a triazole unit (compounds **6d**–**g**) does not afford any positive interaction with aminoacidic residues in the NNRTIs binding pocket. In particular, docking experiments show that the triazole unit induces a different binding mode with loss of hydrogen bonds between the nucleobase and the Lys101. Only compound **6g** shows a modest anti-HIV activity.

According to the presence of the phosphonic unit at C-5′, compound **7** could be regarded as monophosphonylated homo-*N*,*O*-nucleosides, which could be further explored as possible NRTI chain terminators after conversion by phosphorylation into the triphosphate form. The cell-free conditions of the RT inhibition assay employed here did not provide enzymatic phosphorylation. Thus, we determined their ability to inhibit the reverse transcriptase activity by means of a cell-free assay [36], after incubation with a crude extract from 1 × 10^6^ PBMCs (human peripheral blood mononuclear cells), which serves as enzyme supplier for phosphorylation processes. Zidovudine (AZT) (a well-known nucleoside) and tenofovir (a phosphonated nucleoside actually used in antiviral chemotherapy) were utilized in the assay as internal positive controls. The obtained results indicate that compound **7b** exhibits a possible inhibition of RT HIV in the range 85–120 μM: the R-anomer **7a** being completely inactive at the higher concentration tested (200 μM).

The results obtained from RT inhibitory assays and docking studies suggest that phosphonated reverse homo-*N*,*O*-nucleosides **6a**–**c** represent an interesting class of biologically active molecules, worthy of investigation by SAR and in vivo toxicology studies in order to obtain new anti-HIV agents with a good pharmacological profile.

## 3. Materials and Methods

### 3.1. General Information

Solvents and reagents were used as received from commercial sources. Melting points were determined with a Kofler apparatus. HRMS were determined with a TSQ Quantum XLS Triple Quadrupole GC-MS/MS (Thermo Scientific, Waltham, MA, USA). NMR spectra (^1^H-NMR recorded at 500 MHz, ^13^C-NMR recorded at 125 MHz) were obtained in CDCl_3_ solution on a Varian instrument (Agilent Technologies, Palo Alto, CA, USA), and data are reported in ppm relative to TMS as an internal standard. Thin-layer chromatographic separations were carried out on Merck silica gel 60-F254 precoated aluminum plates (Merck, Darmstadt, Germany). Flash chromatography was carried out using Merck silica gel (200–400 mesh). Preparative separations were carried out using a Büchi C-601 MPLC instrument (BUCHI Italia S.r.l., Milano, Italy) using Merck silica gel 0.040–0.063 mm, and the eluting solvents were delivered by a pump at the flow rate of 3.5–7.0 mL/min. All solvents were dried according to methods in the literature. The identification of samples from different experiments was secured by mixed melting points and superimposable NMR spectra.

Compounds **8–10** were synthesized as described previously [48]. 5-Substituted uracils **11a**–**c** were prepared by reaction of 5-hydroxymethyluracil with allylic, propargylic and benzylic alcohols, respectively, in conc. HCl at reflux. Azides **12a**–**d** were synthesized by two different procedures [59]: Compounds **12a**,**b** were obtained by treatment of substituted anilines with sodium nitrite and an equimolar amount of sodium azide in acetonitrile, at 0–5 °C for 1 h; and compounds **12c**,**d** were prepared starting from the corresponding benzyl halides, by treatment with sodium azide and ammonium chloride [67].

Physical and spectroscopic data for compounds **7a**,**b** are superimposable with that reported in literature [61].

C-[(tert-butyldiphenylsilyl)oxy]-N-methyl nitrone **13** and isoxazolidines **14** were prepared as reported previously [62,63].

### 3.2. General Procedure for the Preparation of 3-Pyrimidinyl-Isoxazolidines ***6a**–**c***

A suspension of 5-substituted uracils **11a**–**c** (0.84 mmol) in anhydrous CH_3_CN (3 mL) was treated with bis(trimethylsilyl)acetamide (BSA; 2.52 mmol) and was stirred until the solution was clear (15 min). A solution of benzyl 3-acetoxyisoxazolidine-2-carboxylate (**10**; 0.56 mmol) in anhydrous CH_3_CN (3 mL) and trimethylsilyl triflate (TMSOTf; 0.11 mmol) was then added and the reaction was heated at 70 °C for 5 h. After cooling to 0 °C, the solution was carefully neutralized by addition of 5% aq. NaHCO_3_ and then concentrated in vacuo. CH_2_Cl_2_ (30 mL) was added and the organic phase was separated, washed with water (2 × 10 mL), dried (Na_2_SO_4_), filtered and evaporated to dryness. The residue was purified by MPLC on a silica gel column (CH_2_Cl_2_/MeOH, 98:2) to afford the desired compound (60%–70%).

### 3.3. General Procedure for the Preparation of 3-Pyrimidinyl-Isoxazolidines ***6d**–**g***

To a solution of **6c** (1.0 eq.), aryl/alkyl azides **12a**–**d** (1.1 eq.) and triethylamine (1.0 eq.) in *tert*-BuOH (40 mL) and H_2_O (40 mL), CuSO_4_·5H_2_O (0.25 eq.) and sodium ascorbate (0.5 eq.) were added. The mixture was allowed to stir at room temperature for 4 h under N_2_ and concentrated. The mixture was then diluted with water and extracted with ethyl acetate (3 × 30 mL). The organic layer was dried over MgSO_4_, filtered and concentrated at reduced pressure and the residue was purified by flash chromatography. (CH_2_Cl_2_/MeOH, 98:2). The compounds were crystallized in diethyl ether/CH_2_Cl_2_.

## 4. Conclusions

New pyrimidine-2,4-diones linked to an isoxazolidine nucleus have been synthesized and tested as compounds endowed with potential anti-HIV activity. Compounds **6a**–**c**, characterized by the presence of an ethereal substituent at C-3, showed interesting biological features, acting as NNRTIs, with compound **6c** showing the highest HIV-RT inhibitor activity in the nanomolar range, and compound **6b** the highest inhibitory effect on HIV-1 infection with no toxicity. The lack of cytotoxicity and a possible anti-HIV-1 activity indicates the new compounds **6a**–**c** as interesting starting points for future investigations. 

Conversely, compounds **6d**–**g** did not show any HIV RT inhibitory activity, nor an ability to decrease HIV infection in CEM-GFP cells and exhibited higher levels of cytotoxicity than **6a**–**c**. Interestingly, compound **6g** was the only one of this group of compounds able to inhibit in vitro infection with HIV-1 of CEM-GFP cells at 103 µM concentration. This finding suggests a slight activity of **6g** on HIV replication, presumably through mechanisms other than RT inhibition, although the SI is too low in comparison with **6a**–**c**. This might be related to the presence of the alcoholic group. Further studies are necessary to clarify this point.

Compound **7b**, monophosphonylated homo-*N*,*O*-nucleoside, where the heterocyclic base is linked at the C-3′ of the isoxazolidine unit by a methylene linker, showed only a slight inhibition of RT HIV, acting as an NRTI chain terminator.

The obtained data clearly indicate that the pyrimidine-2,4-dione unit deserves further investigation as a valuable scaffold for extending the current spectrum of antiviral activity of modified nucleosides, avoiding some unwanted side effects. SAR and in vivo toxicology studies performed on phosphonated reverse homo-*N*,*O*-nucleosides **6a**–**c** could allow the development of new anti-HIV agents with a better pharmacological profile than that exerted by the currently used anti-HIV agents.

## Figures and Tables

**Table 1:** Inhibitory activities against human immunodeficiency virus (HIV)-1 reverse transcriptase (RT) of pyrimidinylisoxazolidines and their cytotoxicity.

	RT Inhibition Assay ^1^ MRTIC (μM)	Cytotoxicity CC50 ± SD (μM) ^2^	
Compound	HIV-RT	MOLT-3	Pearson’s ^3^
**6a**	0.1	>1000	0.94
**6b**	1	>1000	0.95
**6c**	0.01	>1000	0.97
**6d**	>100	594 ± 0.8	-
**6e**	>100	579 ± 2	-
**6f**	>100	552 ± 3	-
**6g**	100	670 ± 1	-
**7a**	n.d.	>1000	-
**7b**	90 ^4^	>1000	-
**NVP**	0.001	>1000	0.97
**RPV**	0.038	n.d.	n.d.
**EVF**	0.0000094	n.d.	n.d.

^1^ Minimal inhibitory concentration (MIC) against HIV-RT, defined as the minimum concentration required to completely inhibit (100%) RT, was evaluated in a cell-free assay. The compounds were tested in two rounds. In the first round, the concentration range was between 200 and 1 μM. In the second round, compounds were tested at concentrations between 1 and 0.001 μM. ^2^ Half-maximal cytotoxic concentration (CC_50_), defined as the concentration required to decrease metabolic activity by 50% ± standard deviation (SD), was evaluated in MOLT-3 cells using an MTS assay. ^3^ The Pearson product–moment correlation coefficient (Pearson’s *r*), which reflects the degree and direction of the linear relationship between two variables, was calculated for each significant CC_50_ value. ^4^ After incubation with a crude extract from PBMCs (human peripheral blood mononuclear cells).

**Table 2:** Inhibitory activities against human immunodeficiency virus (HIV)-1 infection assayed by flow cytometry analysis of HIV-1 infected CEM cells harboring a plasmid of the green fluorescence protein under the control of HIV-LTR (CEM-GFP).

	IC50 ± SD (µM) ^1^	Cytotoxicity CC50 ± SD (µM) ^2^	Selectivity Index ^3^
Compound	% GFP + cells	CEM/GFP	SI
**6a**	10.1	747 ± 10.7	73.9
**6b**	0.69	1053 ± 8.2	1526
**6c**	15.7	1127 ± 6.6	71.8
**6g**	103	427 ± 0.1	4
**NVP**	0.14	980 ± 9.9	7000

^1^ Inhibitory concentration 50 (IC50), defined as the compound and or drug concentration required to decrease by 50% HIV-1 infection of CEM-GFP+ cells.^2^ Half-maximal cytotoxic concentration (CC50), defined as the concentration required to decrease metabolic activity by 50% ± standard deviation (SD), as evaluated in uninfected CEM-GFP cells using an MTS assay.^3^ Selectivity index: CC50/IC50

**Table 3:** ΔG of binding of compounds **6a**–**c**.

Compound	ΔG Kcal/mol
**6a**	−8.51
**6b**	−7.45
**6c**	−8.62

## Supplementary Materials

Copies of ^1^H-NMR spectra of all new compounds; protocols for inhibition assays.
