# Supplementary material for: Pyrimidine 2,4-Diones in the Design of New HIV RT Inhibitors
Source: Molecules. 2019 May 2;24(9):1718. doi: 10.3390/molecules24091718 (PMC6539630; doi:10.3390/molecules24091718)
Supplement: Supplementary file 1 [file molecules-24-01718-s001.pdf]

Supporting Information

Pyrimidine 2,4-diones in the Design of New HIV RT Inhibitors

Roberto Romeo[1,*], Daniela Iannazzo[2], Lucia Veltri[3], Bartolo Gabriele[3], Beatrice Macchi[4], Caterina Frezza[4], Francesca Marino-Merlo[5] and Salvatore V. Giofrè[1,*]

[1] Dipartimento di Scienze chimiche, biologiche, farmaceutiche ed ambientali, Università di Messina, Via S.S. Annunziata, 98168 Messina, Italy
[2] Dipartimento di Ingegneria, Università di Messina, Contrada Di Dio, 98166 Messina, Italy; diannazzo@unime.it (D.I.)
[3] Dipartimento di Chimica e tecnologie chimiche, Università della Calabria,Via P. Bucci 12/C, 87036 Arcavacata di Rende, Italy; lucia.veltri@unical.it (L.V.); bartolo.gabriele@unical.it (B.G.)
[4] Dipartimento di Scienze e Tecnologie Chimiche, Università di Roma "Tor Vergata", 00133 Roma, Italy; macchi@med.uniroma2.it (B.M.); catafrezza@hotmail.it (C.F.)
[5] IRCCS Centro Neurolesi "Bonino-Pulejo", Messina, Italy; fmarino@unime.it

Table of Contents

Experimental data of compounds **6a-g**.. S3

Biological assay..S10

Computational methods..S11

Benzyl 3-(2,4-dioxo-5-((allyloxy)methyl)-3,4-dihydropyrimidin-1(2H)-yl)isoxazolidine-2-carboxylate 6a: White solid (70 % yield): mp: 141–143 °C; ^{1}H NMR (500 MHz, $CDCl_3$): = 8.43 (br s, 1H), 7.52 (s, 1H), 7.41–7.31 (m, 5H), 6.65 (dd, *J*= 4.5, 8.2 Hz, 1H), 5.97–5.89 (ddd, *J*=6.0, 10.2, 17.3 Hz, 1H), 5.25 (s, 2H), 5.36–5.15 (ddd, *J*=1.4, 10.2, 17.3 Hz, 2H), 4.32–4.19 (m, 3H), 4.10–4.02 (m, 2H), 3.93–3.88 (m, 1H), 3.03–2.93 (m, 1H), 2.39–2.25 (m, 1H); ^{13}C NMR (125 MHz, $CDCl_3$): =37.3, 64.2, 69.1, 70.1, 70.3, 72.1, 112.7, 117.8, 128.1, 128.5, 128.8, 134.2, 134.8, 136.9, 149.8, 156.0, 162.0. HRMS (ESI+): m/z $[M+H]^+$ calcd for $C_{19}H_{22}N_3O_6$: 388,1509, found: 388.1512.

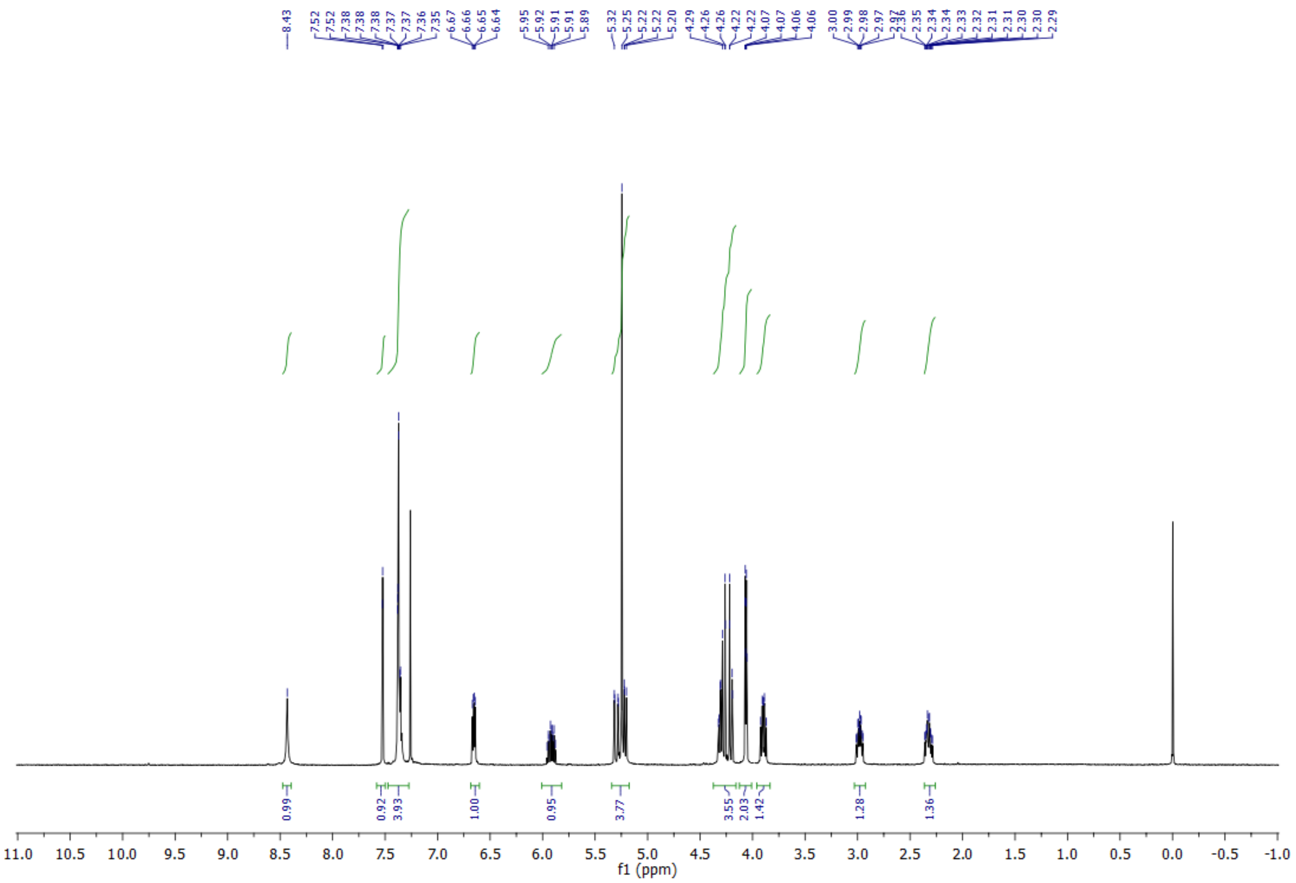

Benzyl 3-(2,4-dioxo-5-((prop-2-yn-1-yloxy)methyl)-3,4-dihydropyrimidin-1(2H)-yl)isoxazolidine-2-carboxylate 6b: Pale yellow solid (80 % yield): mp: 125-127°C; ^{1}H NMR (500 MHz, $CDCl_3$) δ = 9.07 (brs, 1H),

7.53 (s, 1H), 7.44 – 7.28 (m, 5H), 6.65 (dd, *J* = 8.2, 4.5 Hz, 1H), 5.24 (s, 2H), 4.39 – 4.27 (m, 3H), 4.23 (s, 2H), 3.89 (dd, *J* = 16.9, 9.0 Hz, 1H), 3.02 – 2.94 (m, 1H), 2.46 (t, *J* = 2.4 Hz, 1H), 2.36 – 2.25 (m, 1H).^{13}C NMR (125 MHz, $CDCl_3$): =37.5, 58.2, 64.1, 69.1, 70.1, 70.3, 75.2, 112.0, 128.5, 128.7, 128.8, 134.8, 137.4, 149.9, 156.0, 162.2. HRMS (ESI+): m/z $[M+H]^+$ calcd for $C_{19}H_{20}N_3O_6$: 386,1352, found: 386,1356.

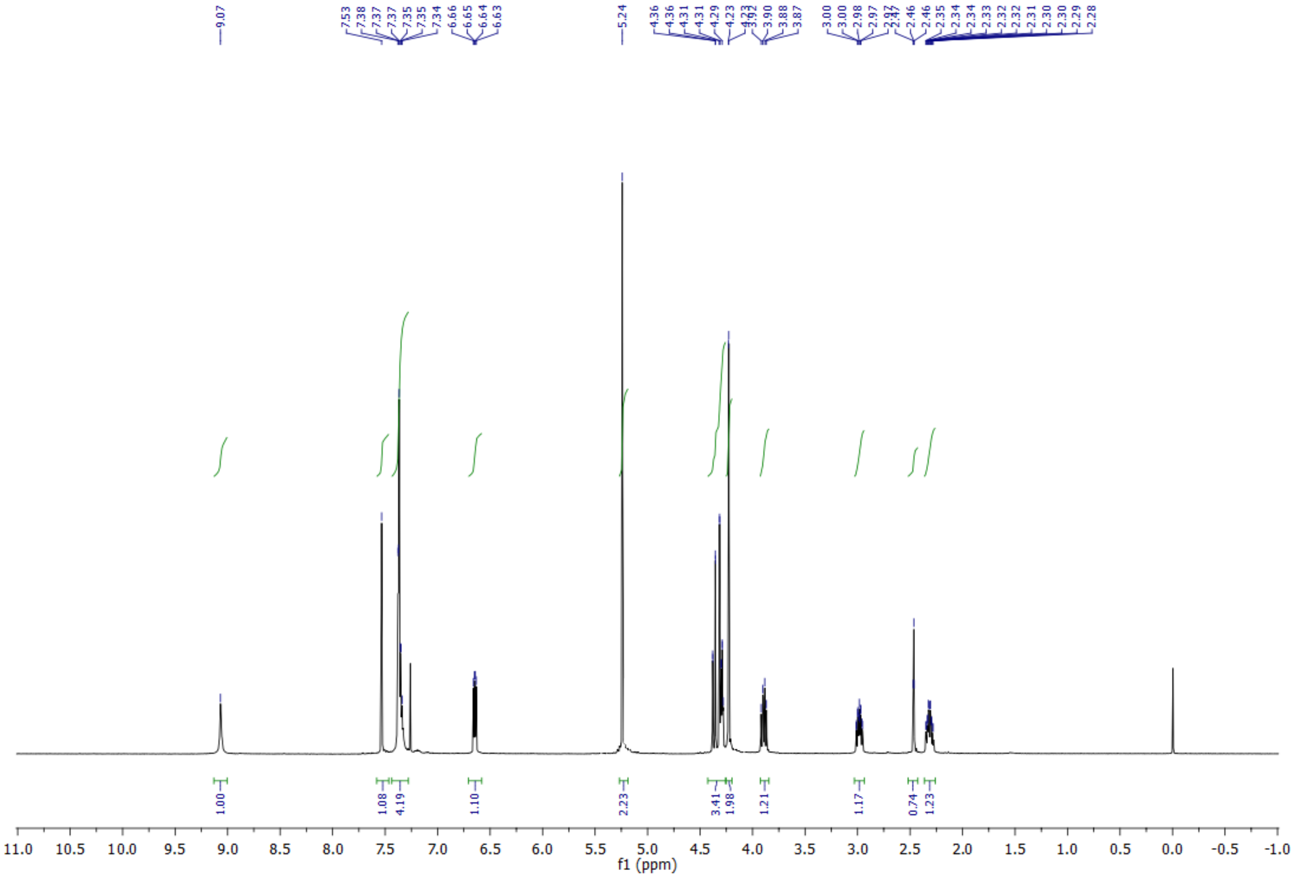

Benzyl 3-(2,4-dioxo-5-((benzyloxy)methyl)-3,4-dihydropyrimidin-1(2H)-yl)isoxazolidine-2-carboxylate 6c:White solid (75 % yield): mp: 150-151 °C; ^{1}H NMR (500 MHz, $CDCl_3$): = 8.14 ppm (br s, 1H), 7.37 (s, 1H),

7.35–7.27 (m, 10H), 6.65 (dd, *J* = 8.2, 4.5 Hz, 1H), 5.25 (s, 2H), 4.60 (s, 2H), 4.30–4.25 (m, 3H), 3.90 (dt, *J* = 8.6, 8.0 Hz, 1H), 2.99–2.94 (m, 1H), 2.35 –2.27 (m, 1H);^{13}C NMR (125 MHz, $CDCl_3$): = 37.5, 64.4, 69.1, 70.1, 70.3, 73.2, 112.7, 127.9, 128.5, 128.7, 128.8, 134.8, 137.0, 137.6, 149.8, 156.0, 162.0. HRMS (ESI+): m/z $[M+H]^+$ calcd for $C_{23}H_{24}N_3O_6$: 438,1665, found: 438,1667.

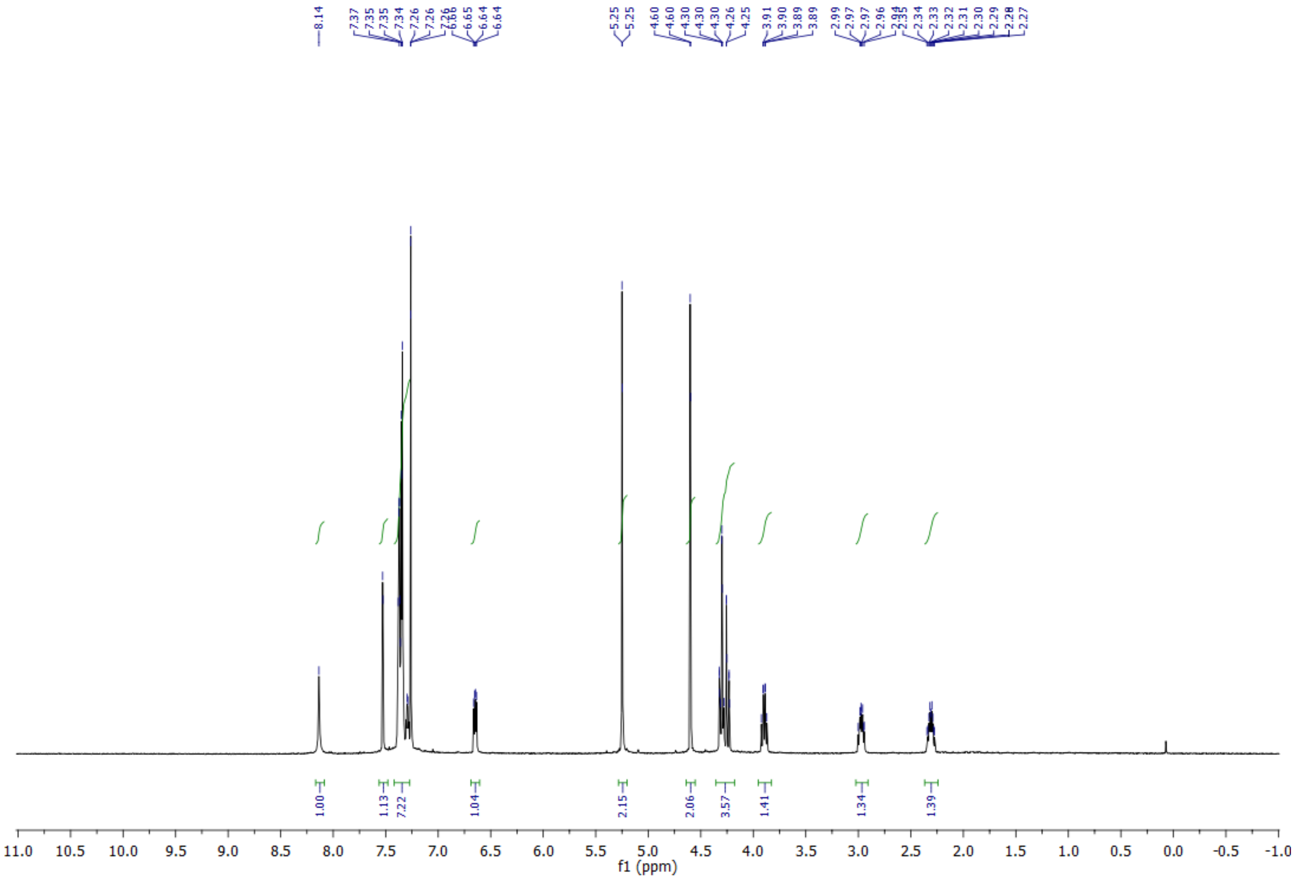

Benzyl 3-(5-(((1-(4-methoxyphenyl)-1H-1,2,3-triazol-4-yl)methoxy)methyl)-2,4-dioxo-3,4-dihydropyrimidin-1(2H)-yl)isoxazolidine-2-carboxylate 6d. White solid (84 % yield): mp: 155-156 °C; ^{1}H NMR (500 MHz, $CDCl_3$) δ = 8.40 (bs, 1H), 7.99 (s, 1H), 7.63 (d, *J* = 8.9 Hz, 2H), 7.58 (s, 1H), 7.39 – 7.29 (m, 5H), 7.01 (d, *J* = 8.9 Hz, 3H), 6.66 (dd, *J* = 8.1, 4.5 Hz, 1H), 5.23 (s, 2H), 4.78 (s, 2H), 4.44 – 4.27 (m, 3H), 3.93 – 3.82 (m, 4H), 3.01 – 2.92 (m, 1H), 2.42 – 2.30 (m, 1H). ^{13}C NMR (125 MHz, $CDCl_3$) δ = 162.09, 159.82, 155.97, 149.76, 137.89, 128.75, 128.68, 128.44, 122.20, 114.76, 70.23, 69.14, 64.66, 64.17, 55.61, 37.43, 29.67. HRMS (ESI+): m/z $[M+H]^+$ calcd for $C_{26}H_{27}N_6O_7$: 535,1941, found 535,1945.

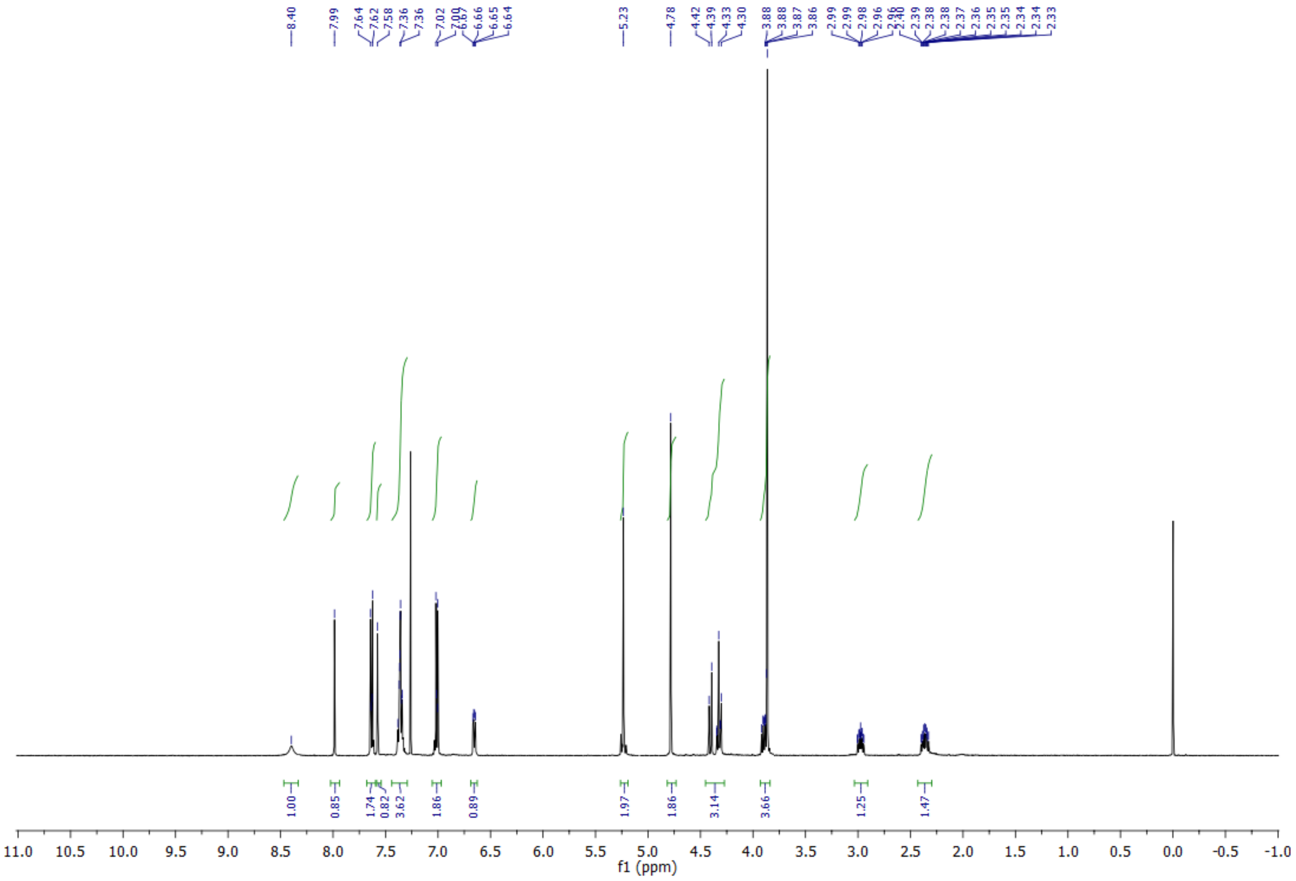

Benzyl 3-(5-(((1-(4-fluorophenyl)-1H-1,2,3-triazol-4-yl)methoxy)methyl)-2,4-dioxo-3,4-dihydropyrimidin-1(2H)-yl)isoxazolidine-2-carboxylate 6e. Yellow solid (80 % yield): mp: 148-149 °C; ^{1}H NMR (500 MHz, $CDCl_3$) δ = 8.83 (bs, 1H), 8.06 (s, 1H), 7.76 – 7.68 (m, 2H), 7.58 (s, 1H), 7.43 – 7.30 (m, 5H), 7.20 (t, *J* = 8.5 Hz, 2H), 6.66 (dd, *J* = 8.0, 4.4 Hz, 1H), 5.24 (s, 2H), 4.79 (s, 2H), 4.43 – 4.25 (m, 3H), 3.94 – 3.84 (m, 1H), 3.02 – 2.92 (m, 1H), 2.40 – 2.26 (m, 1H). ^{13}C NMR (125 MHz, $CDCl_3$) δ = 162.46, 149.89, 138.00, 134.73, 128.77, 128.69, 128.45, 122.53 (d, *J* = 8.6 Hz), 121.25, 116.77, 116.59, 112.01, 70.29, 70.17, 69.18, 64.68, 64.11, 37.48. HRMS (ESI+): m/z $[M+H]^+$ calcd for $C_{25}H_{24}FN_6O_6$: 523,1741, found 523,1744.

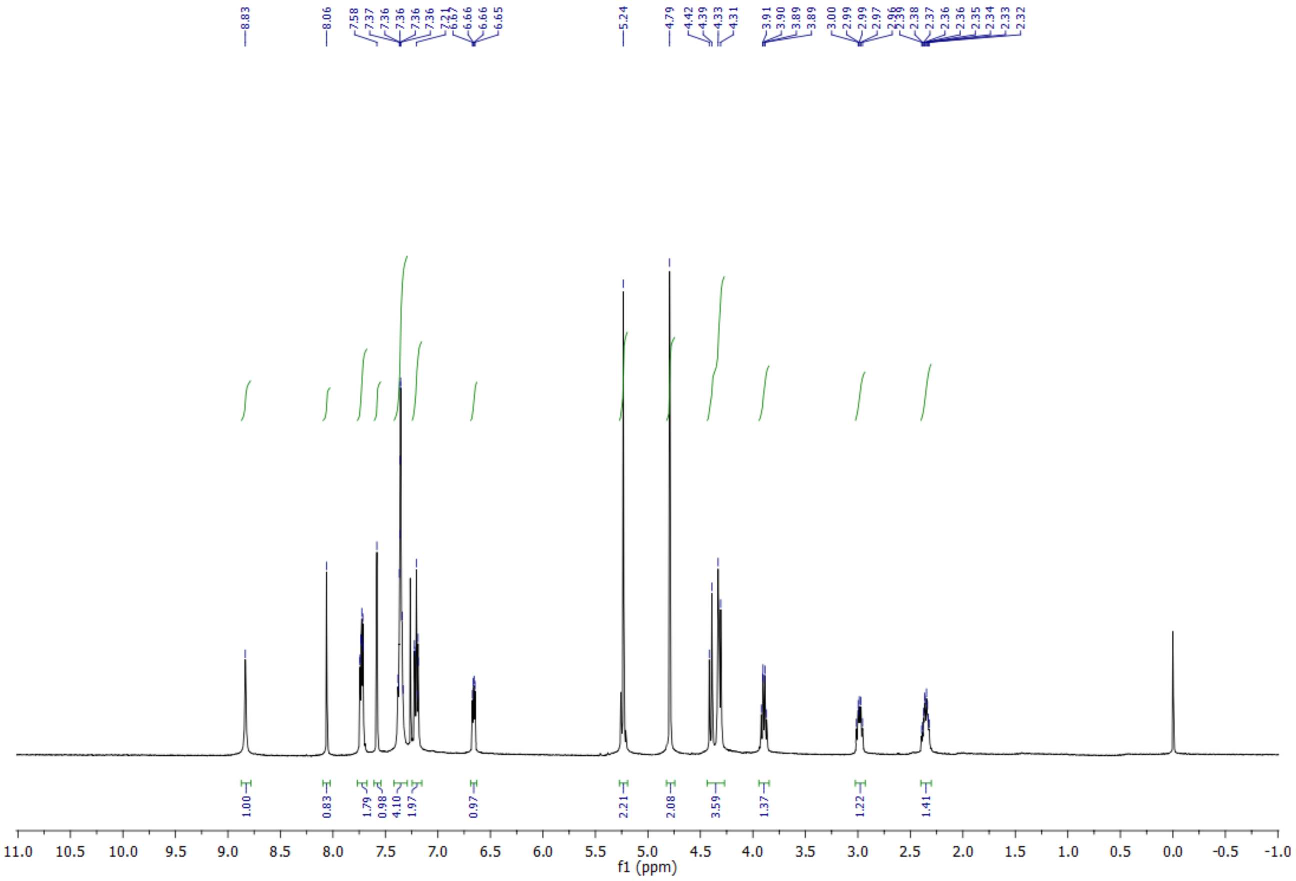

Benzyl 3-(5-(((1-benzyl-1H-1,2,3-triazol-4-yl)methoxy)methyl)-2,4-dioxo-3,4-dihydropyrimidin-1(2H)-yl)isoxazolidine-2-carboxylate 6f. White solid (90 % yield): mp: 145-146 °C; ^{1}H NMR (500 MHz, $CDCl_3$) δ = 8.84 (bs, 1H), 7.54 (s, 1H), 7.52 (s, 1H), 7.39 – 7.27 (m, 10H), 6.64 (dd, *J* = 8.1, 4.4 Hz, 1H), 5.51 (s, 2H), 5.23 (s, 2H), 4.68 (s, 2H), 4.36 – 4.21 (m, 3H), 3.87 (dd, *J* = 17.3, 8.6 Hz, 1H), 2.99 – 2.91 (m, 1H), 2.38 – 2.28 (m, 1H). ^{13}C NMR (125 MHz, $CDCl_3$) δ = 162.25, 149.84, 137.82, 134.78, 134.55, 129.08, 128.86, 128.60, 128.44, 128.13, 122.77, 112.08, 70.15, 69.13, 64.58, 64.17, 54.14, 37.39. HRMS (ESI+): m/z $[M+H]^+$ calcd for $C_{26}H_{27}N_6O_6$: 519,1992, found 519,1996.

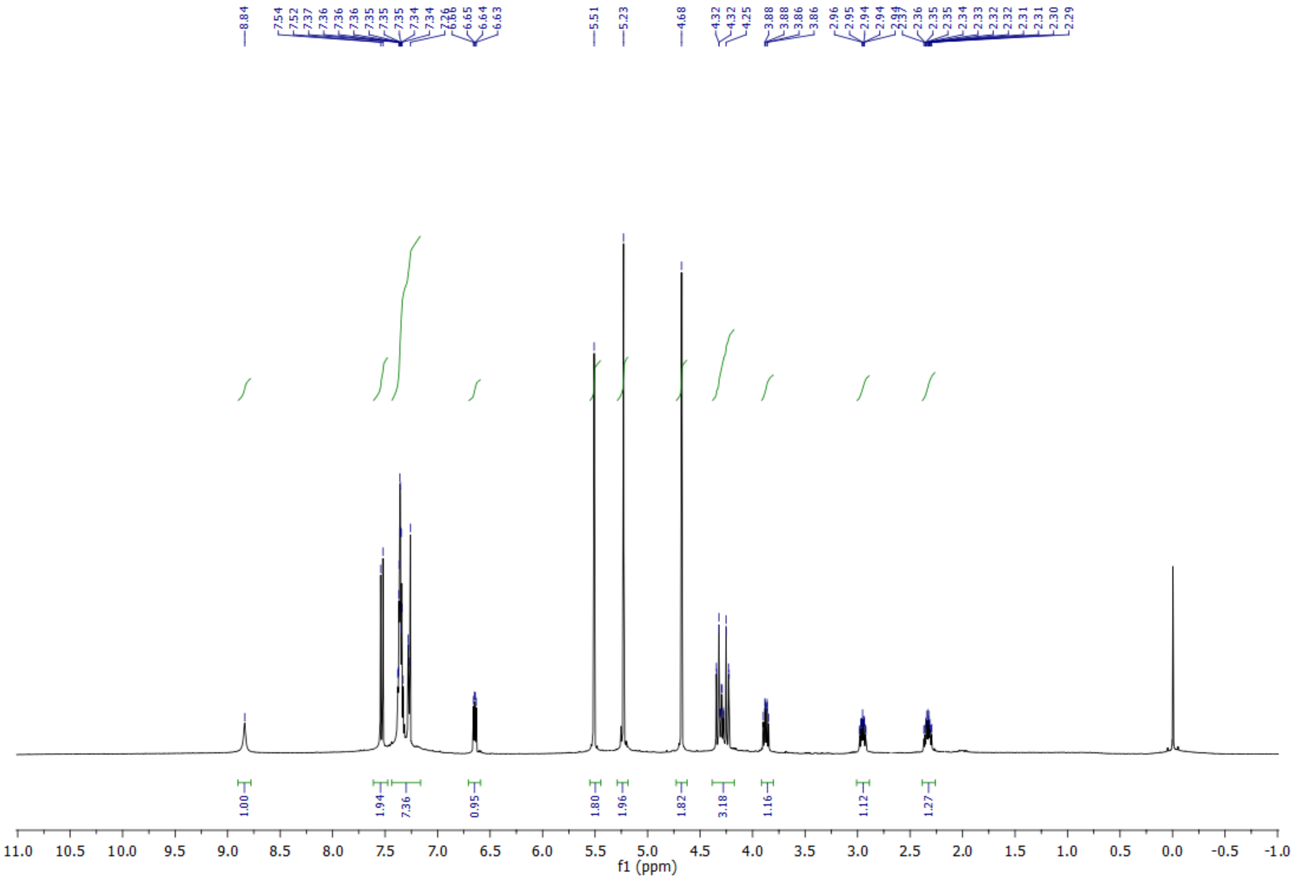

Benzyl 3-(5-(((1-(2-hydroxyethyl)-1H-1,2,3-triazol-4-yl)methoxy)methyl)-2,4-dioxo-3,4-dihydropyrimidin-1(2H)-yl)isoxazolidine-2-carboxylate 6g. White solid (82 % yield): mp: 141-142 °C; ^{1}H NMR (500 MHz, $CDCl_3$) δ = 9.42 (bs, 1H), 7.80 (s, 1H), 7.54 (s, 1H), 7.37 – 7.32 (m, 5H), 6.63 (dd, *J* = 8.0, 4.3 Hz, 1H), 5.23 (s, 2H), 4.70 (s, 2H), 4.48 (dd, *J* = 6.3, 3.5 Hz, 2H), 4.32 – 4.18 (m, 3H), 4.04 – 3.97 (m, 2H), 3.88 (ddd, *J* = 10.1, 8.6, 7.3 Hz, 1H), 3.31 (bs, 1H), 3.00 – 2.93 (m, 1H), 2.39 – 2.30 (m, 1H). ^{13}C NMR (125 MHz, $cdcl_3$) δ = 156.01, 150.03, 138.36, 134.74, 128.76, 128.69, 128.45, 70.24, 69.18, 64.21, 61.16, 52.69, 37.40, 29.67. HRMS (ESI+): m/z $[M+H]^+$ calcd for $C_{21}H_{25}N_6O_7$: 473,1785, found 473,1789.

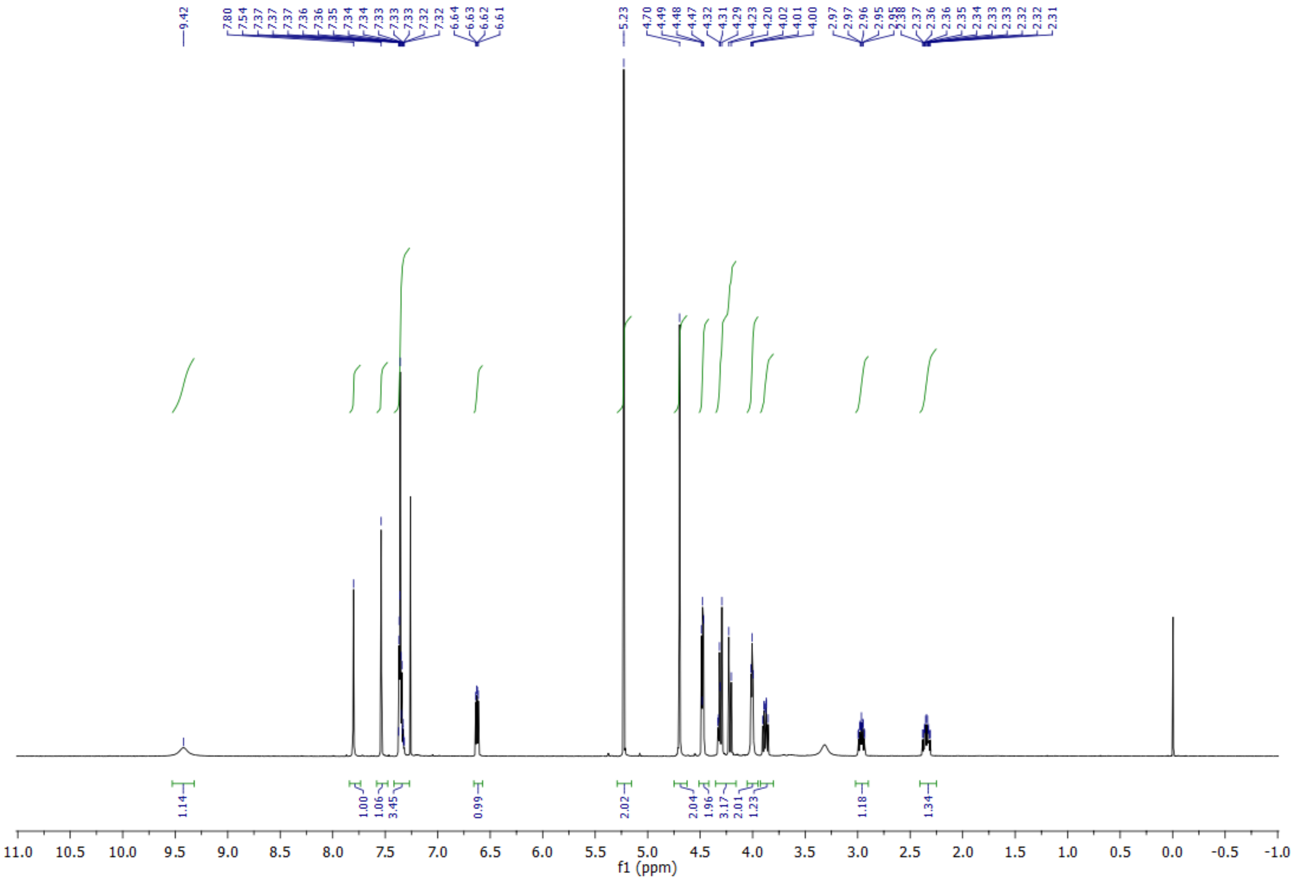

Biological assays

Cytotoxic assay

The cytotoxicity of the compounds was evaluated by a commercial colorimetric kit MTS (3-(4,5-dimethylthiazol-2-yl)-5-(3-carboxymethoxyphenyl)-2-(4-sulfophenyl)-2H-tetrazolium) (Cell Titer 96 Aqueous One Solution, Promega, Madison,WI). Inhibition of cell metabolic activity was detected by reduction of the MTS to formazan. The assay was performed by seeding 6 x 10^4 for CEM and/or U937 cells in the presence or absence of the different compounds at concentrations ranging from 1000 to 0.01 μM. Nevirapine was used as an internal positive control, at the concentrations of 1, 0.1 and 0.01 μM. After 24 hours, 20 μl of MTS were added directly to culture wells and samples were incubated for 1 h. Successively the absorbance was read at 490 nm. Results were expressed as the drug concentration required to inhibit 50% of the metabolic activity (CC_{50}).

RT assay

The RT-PCR assay was performed as previously described [19a]. Briefly, RNA isolated from gD expressing transfectant cells was used as a template. Isolation of total RNA from transfectant cells was performed using Nucleo Spin RNA II (Macherey–Nagel, Duren, Germany) according to the manufacturer's instructions and dissolved in diethylpyrocarbonate (DPEC)-treated distilled water. Total RNA (150 ng) was reverse transcribed using US6 0.5 mM reverse primer (5′-TGT CGT CAT AGT GGG CCT CCA T-3′) in a reaction mixture containing 1 U recombinant HIV-RT (Invitrogen, Carlsbad, CA) and/or 1 U AMV, 1x RT buffer, 0.2mM dNTP (all from Promega, Madison, WI), in the presence or absence of the drugs at different concentrations in a final volume of reaction mixture of 25 ml for 1 hr at 37°C. The compounds were tested in two rounds. In the first round, the concentration range was between 1000 and 1 μM. In the second round, compounds were tested at concentrations between 1 and 0.001 μM. Nevirapine was assayed at the concentrations of 1,0.1 and 0.01 μM. After incubation at 80°C for 5 m, cDNA was used for DNA PCR. Amplification was performed in a reaction mixture containing 2 ml cDNA, 0.5 mM primers (US6 reverse 50-TGT CGT CAT AGT GGG CCT CCA T-30) and (US6 forward primer 50-AGA CTT GTT GTA GGA GCA TTC G-30), 1mM dNTP, 1x Taq buffer, 5mM MgCl2, and 0.5U Taq DNA polymerase (Fisher Scientific, Illkirch Cedex, France), for 35 cycles (30 sec at 95°C, 30 sec at 59°C, and 45 sec at 72°C) on a Master cycler (Eppendorf, Hamburg, Germany). Following the final cycle, samples were incubated at 72°C for 20 min to ensure the completion of the final extension step. Amplified DNA (350 bp) was visualized on 1.5% agarose gel containing 10 mg/ml ethidium bromide in 1 TAE buffer. As a marker, a 100 bp DNA ladder was used (New England Biolabs),

HIV infection assay

We assessed the ability on these compounds to inhibit the infection of CEM –GFP cells with HIV [19b]. Briefly CEM-GFP cells (1%105 cell) were resuspended in 50 of RPMI 1640 plus 10% FBS complete medium (CM) and unexposed or exposed to several concentrations of compounds (100,50,25,12.5 μM) or to drug Nevirapine (1,0.1 and 0.01 μM) for 2hr at 37°C in a humidified atmosphere supplemented with 5% CO2. Virus was added at 300ng, and the plates were subjected to spinoculation and incubated for 3 h at 37 °C. Excess of virus was

removed and 500 µl of CM alone or containing the same amount of compounds was added. Infection was determined by assessing GFP expression through cytofluorimetric analysis after 72 hours of incubation.

Computational methods

All molecular modeling calculations and manipulations were performed with software packages Moe 2008.10 and AutoDock 4.2, running on Red Hat Linux CentOs workstations. The determination of ligand interaction features for each pose within the binding pocket of receptor were analyzed by LigandScout Advanced 4.1 Inte:Ligand GmbH, Vienna, Austria.

Docking studies. The structures of **6a-g**, were built using the Maestro 3D-sketcher and fully minimized (Polak-Ribiere conjugate gradient, 0.05 kJ/Å·mol convergence). Atom charges assigned to compounds during the minimization step were retained for the following docking calculations. Complexes generated by docking simulations were then refined with Moe. For the docking procedure, the program AutoDock 4.2 was used to explore the binding conformation of **6a-g** on the wt and/or mutated RT. A grid spacing of 0.375 Å and 60 x 60 x 60 number of points was set, comprising all residues that constitute the NNRTI binding pocket. At each grid point, the receptor's atomic affinity potentials for carbon, oxygen, nitrogen, sulfur, and hydrogen were precalculated for rapid intra- and intermolecular energy evaluation of the docking solutions for each inhibitor. The genetic algorithm-local search (GA-LS) method was used with the default settings and retrieved 100 docked conformations from each compound. Results from Autodock calculations were clustered with a root mean square deviation (rmsd) tolerance of 1.5 Å and the lowest energy conformer of the most populated cluster (the lowest energy cluster in most cases) was selected as the most probable binding conformer.
